# Critical Needs in Advancing *Shigella* Vaccines for Global Health

**DOI:** 10.1093/infdis/jiab462

**Published:** 2021-09-24

**Authors:** Calman A MacLennan, Kawsar R Talaat, Robert W Kaminski, Dani Cohen, Mark S Riddle, Birgitte K Giersing

**Affiliations:** 1 Bill & Melinda Gates Foundation, London, United Kingdom; 2 Center for Immunization Research, Department of International Health, Johns Hopkins Bloomberg School of Public Health, Baltimore, Maryland, USA; 3 Diarrheal Disease Research, Bacterial Diseases Branch, Walter Reed Army Institute of Research, Silver Spring, Maryland, USA; 4 School of Public Health, Sackler Faculty of Medicine, Tel Aviv University, Tel Aviv, Israel; 5 University of Nevada, Reno School of Medicine, Reno, Nevada, USA; 6 World Health Organization, Geneva, Switzerland

**Keywords:** CHIM, global health, LMICs, *Shigella*, vaccines

## Abstract

Advancing new O-antigen-based *Shigella* vaccines is critically dependent on development of an international standard serum and harmonized ELISA, demonstration of field efficacy in young children in low- and middle-income countries, and early engagement with regulators and policy makers.

New O-antigen-based vaccines against *Shigella* are in clinical development. Historical efficacy studies identify serum O-antigen immunoglobulin G as a correlate of protection, leading to the suggestion that accelerated licensure could be achieved using the *Shigella*-controlled human infection model (CHIM). We discuss the role of the *Shigella* CHIM alongside critical imperatives needed for accelerated licensure and uptake of *Shigella* vaccines: (1) development of an international standard serum and harmonized enzyme-linked immunosorbent assay; (2) demonstration of field efficacy in young children in low- and middle-income countries; and (3) early engagement with regulators and policy makers.

*Shigella* is a major cause of diarrhea and dysentery disproportionately affecting children in low- and middle-income countries (LMICs) and responsible for 164 300 deaths annually [1]. A World Health Organization (WHO) expert consultation on *Shigella* vaccine preferred product characteristics concluded the following: “the primary goal is to develop a safe, effective and affordable vaccine to reduce mortality and morbidity due to dysentery and diarrhea caused by *Shigella* in children under 5 years of age in LMICs” [2]. A vaccine would also benefit travelers. *Shigella* species express different O-antigens. A vaccine will need to protect against *Shigella sonnei* and *Shigella flexneri* 2a, 3a, and 6 to cover the most prevalent globally circulating strains [3].

Over 20 years ago, a National Institutes of Health (NIH) glycoconjugate vaccine of *S sonnei* O-antigen conjugated to recombinant exotoxin of *Pseudomonas aeruginosa* (rEPA) conferred 74% protection against *S sonnei* shigellosis in Israeli military [4], protection associating with serum immunoglobulin G (IgG) to O-antigen. The vaccine also protected children aged 3 to 4 years but not children under 3 years [5]. Loss of protection associated with decreasing serum O-antigen IgG seen with decreasing age. These findings indicate that serum O-antigen IgG is associated with protection, which suggests that a vaccine inducing high IgG levels among LMIC children may confer protection. We define this association as a correlate of protection, in which the attribute, serum O-antigen IgG, is statistically associated with the endpoint, protection against shigellosis, without the association necessarily being causal [6].

*Shigella sonnei*-rEPA used random coupling for conjugation resulting in a heterogenous lattice structure. Variable amounts of free polysaccharide may have interfered with the immunologic response, as seen in mice where admixing free *Streptococcus pneumoniae* type 4 polysaccharide with *S pneumoniae* type 4 polysaccharide-protein conjugate decreased polysaccharide antibody responses [7]. Subsequent vaccine candidates used specific coupling to conjugate O-antigen to carrier protein producing a sun-type structure. This structure with shorter O-antigens induced greater immunogenicity in mice compared with first-generation vaccine [8]. Short saccharides and chemically controlled coupling through a single point on the saccharide improves reproducibility and vaccine characterization. Selective chemistries also avoid modification of the saccharide chain.

Enhanced immunogenicity with reduced saccharide length and site-specific conjugation is seen with dextran conjugates [9]. Pneumococcal saccharide-protein conjugates using synthetic short-chain oligosaccharides and site-specific chemistries are immunogenic and protective in mice [10, 11]. Hence, improved design of *Shigella* conjugates could increase immunogenicity, overcoming lack of protection in young children. A *S flexneri* 2a bioconjugate developed by Limmatech (GSK), with O-antigen covalently coupled in sun-type format to rEPA, was immunogenic in US adults [12], and it protected against moderate to severe shigellosis/dysentery in a *Shigella*-controlled human infection model (CHIM) study [13], providing further support for serum O-antigen IgG as a correlate of protection. The quadrivalent vaccine is being tested in Kenya (ClinicalTrials.gov NCT04056117).

The GSK Vaccines Institute for Global Health (GVGH) developed a *Shigella* outer membrane vesicle (OMV) vaccine, the vesicles serving as a delivery vehicle for O-antigen. An *S sonnei* OMV candidate was well tolerated and immunogenic in French, United Kingdom (UK) [14], and Kenyan adults [15], but it failed to protect against shigellosis in a CHIM study [16]. A vaccine consisting of *S flexneri* 2a synthetic O-antigen conjugated to tetanus toxoid induced significantly higher O-antigen IgG levels in Israeli adults than an NIH *S flexneri* 2a O-antigen-rEPA vaccine [17]. It is unknown how immunogenic these new O-antigen-based candidates must be in LMIC young children to confer protection. It is crucial to evaluate immunogenicity in this population, defined as infants and children from 6 to 36 months in LMICs.

By accepting serum O-antigen IgG as a correlate of protection against shigellosis [18], an analysis of immunologic data from field efficacy and CHIM studies can help to identify protective thresholds of O-antigen IgG. Additional information can be obtained from observational studies in target pediatric populations, which examine the association between pre-exposure and/or early acute-stage levels of serum O-antigen IgG with levels after culture-proven shigellosis. Paired sera are required from children before and after exposure to *Shigella*. Possible protection against shigellosis through other mechanisms including mucosal IgA and *Shigella* protein antibodies presents a confounder to this approach. Efforts are underway to confirm serum IgG as a correlate of protection and establish protective thresholds [18], but they are hampered by use of different enzyme-linked immunosorbent assay (ELISA) protocols and reference reagents.

An international standard reference serum and harmonized ELISA are required for comparability between studies. The standard serum should cover each serotype within multivalent vaccine formulations and be generated from vaccinees. Such a serum and ELISA will underpin clinical development of all O-antigen-based *Shigella* vaccines [19]. In 2019, a pilot *Shigella* ELISA collaborative study by the National Institute of Biological Standards and Controls (NIBSC), UK, was a first step towards ELISA harmonization. In 2020, a decision was made to harmonize a *Shigella* ELISA protocol at the Walter Reed Army Institute for Research (WRAIR) and develop a first international standard serum at NIBSC with the goal of submitting it to the WHO Expert Committee on Biological Standardization for approval and adoption.

*Shigella* CHIMs are conducted in adults in high-income countries (HICs). Therefore, such studies could potentially be used to license new-generation *Shigella* vaccines for travelers [20]. There is precedent, with CHIM studies used to license the Vaxchora cholera vaccine [21] for travelers. As a correlate of protection, serum O-antigen IgG could permit immunobridging from a CHIM study to an immunogenicity study in infants/young children, providing a quicker licensure route than a phase 3 efficacy study [20]. A CHIM supported WHO prequalification of TypBar-TCV typhoid conjugate vaccine for children [22, 23].

However, a typhoid conjugate vaccine was efficacious in young LMIC children 20 years ago [24]. Efficacy has yet to be shown for a *Shigella* vaccine in this population. It is not known whether serum O-antigen IgG is a correlate of protection in LMIC children, nor whether a threshold of protection determined in HICs will be applicable in these children. Therefore, efficacy needs to be demonstrated in this population and can only be achieved with a phase 3 field study. Such a study could confirm correlate of protection status and protective titers for serum O-antigen IgG in this population, enabling subsequent immunobridging and accelerating licensure for other *Shigella* vaccines.

Nevertheless, CHIM studies could assist licensure for children through licensure for travelers, by facilitating earlier use, awareness, and generation of safety data for a *Shigella* vaccine. In addition, CHIM studies may identify serum IgG thresholds of protection, although the artificial nature of infection and low numbers of participants present limitations. Finally, if CHIM for *S flexneri* 3a and 6 become available, they may confirm that *Shigella* vaccines can protect against these serotypes. The low incidence of shigellosis due to these serotypes means it is difficult in a field setting to demonstrate efficacy against them. Moreover, after a successful phase 3 efficacy study in LMIC infants and children, an established correlate and threshold of protection could leverage a large phase 2 study as basis of licensure for subsequent *Shigella* vaccines. If this is acceptable to the regulators, the strategy could accelerate licensure, which reduces the cost of late-stage clinical development.

In conclusion, an international standard serum and harmonized ELISA are needed to advance new-generation global health *Shigella* vaccines, allowing comparability between studies, confirming serum O-antigen IgG as a correlate of protection, and determining thresholds of protection. Currently, almost all data informing correlates and thresholds of protection are from HIC adults and older children. A field-efficacy study among LMIC infants and young children will be critical to advance a first vaccine to licensure, WHO policy recommendation, and prequalification, which are prerequisites for Gavi-supported introduction.

Proactive scenario planning of regulatory strategies by vaccine developers and planning of data to be collected in late-stage product development to support future policy recommendation will help mitigate delays to postlicensure implementation. Early and parallel engagement with LMIC national regulatory authorities and policy makers, as well as global policy and financing bodies, such as WHO and GAVI, is critical before designing pivotal phase 3 efficacy studies. This is the proposed approach of the authors as *Shigella* vaccine candidates advance to phase 3 clinical evaluation.

## Notes

***Disclaimer.*** The authors alone are responsible for the views expressed in this article and they do not necessarily represent the views, decisions or policies of the institutions with which they are affiliated. Material has been reviewed by the Walter Reed Army Institute of Research. There is no objection to its presentation and/or publication. The opinions or assertions contained herein are the private views of the authors, and are not to be construed as official, or as reflecting true views of the Department of the Army or the Department of Defense.

The investigators have adhered to the policies for protection of human subjects as prescribed in AR 70-25. Some authors (R. W. K.) are employees of the U.S. Government. This work was prepared as part of their official duties. Title 17 U.S.C. §105 provides that “Copyright protection under this title is not available for any work of the United States Government.” Title 17 U.S.C. §101 defines a U.S. Government work as a work prepared by a military service member or employee of the U.S. Government as part of that person’s official duties.

***Potential conflicts of interest.*** All authors: No reported conflicts of interest. All authors have submitted the ICMJE Form for Disclosure of Potential Conflicts of Interest.
